# Transcriptional regulation and chromatin dynamics at DNA double-strand breaks

**DOI:** 10.1038/s12276-022-00862-5

**Published:** 2022-10-13

**Authors:** Sunwoo Min, Jae-Hoon Ji, Yungyeong Heo, Hyeseong Cho

**Affiliations:** 1 Department of Biochemistry, Ajou University School of Medicine, Suwon, 16499 Korea; 2 Genomic Instability Research Center, Ajou University School of Medicine, Suwon, 16499 Korea; 3 Department of Biochemistry and Structural Biology, The University of Texas Health San Antonio, Texas, 78229-3000 USA

**Keywords:** Double-strand DNA breaks, DNA damage response

## Abstract

In eukaryotic cells, DNA damage can occur at any time and at any chromatin locus, including loci at which active transcription is taking place. DNA double-strand breaks affect chromatin integrity and elicit a DNA damage response to facilitate repair of the DNA lesion. Actively transcribed genes near DNA lesions are transiently suppressed by crosstalk between DNA damage response factors and polycomb repressive complexes. Epigenetic modulation of the chromatin environment also contributes to efficient DNA damage response signaling and transcriptional repression. On the other hand, RNA transcripts produced in the G1 phase, as well as the active chromatin context of the lesion, appear to drive homologous recombination repair. Here, we discuss how the ISWI family of chromatin remodeling factors coordinates the DNA damage response and transcriptional repression, especially in transcriptionally active regions, highlighting the direct modulation of the epigenetic environment.

## Epigenetics: How chromosome regulators orchestrate DNA repair at active genes

Gene expression gets turned off in response to DNA damage thanks to a family of proteins that regulate how the genome is packaged into chromosomes. A team led by Sunwoo Min and Hyeseong Cho from Ajou University School of Medicine in Suwon, South Korea, review how the presence of DNA double-strand breaks prompts an array of genome-organizing proteins to jump into action, temporarily shutting down the process by which genes are transcribed into RNA. These proteins, known as histone modifiers and chromatin remodelers, then help coordinate the mending of broken DNA to restore genomic integrity before gene expression starts up again. A better understanding of how regulators of chromosome structure orchestrate DNA repair at active genes could lead to new therapeutic strategy for diseases such as cancer.

## Introduction

Eukaryotic transcription, DNA replication, and DNA repair occur simultaneously within the nucleus, requiring coordinated regulation and control in multiple layers of complex mechanisms. DNA damage and repair can occur at any time and at any locus in the nucleus, including loci at which active transcription is taking place. Thus, mechanisms to prevent collision between the DNA transcription and DNA repair machineries are essential to preserve genomic integrity^1–3^. The chromatin context is mainly organized by chromatin remodeling factors, which tighten and loosen the chromatin structure^4^, and histone modifiers, which can turn transcription on or off at damaged loci. The spatial and temporal regulation of histone modifications upon DNA damage mainly depends on both chromatin remodeling factors and histone modifiers.

Here, we briefly summarize the DNA damage response to DNA double-strand breaks (DSBs) and the DNA repair pathway and discuss how transcription is controlled upon DNA damage, particularly emphasizing the role played by chromatin dynamics. We focus on the role of histone modifications and the ISWI family of chromatin remodeling factors in DSB-induced transcriptional silencing and consider the functional implication of transcriptional silencing in the DNA damage response (DDR) and repair. This review highlights the importance of chromatin versatility coordinated by histone modifiers and chromatin remodelers in DSB-induced transcriptional silencing.

### The DNA damage response and DNA repair

**Fig. 1:**
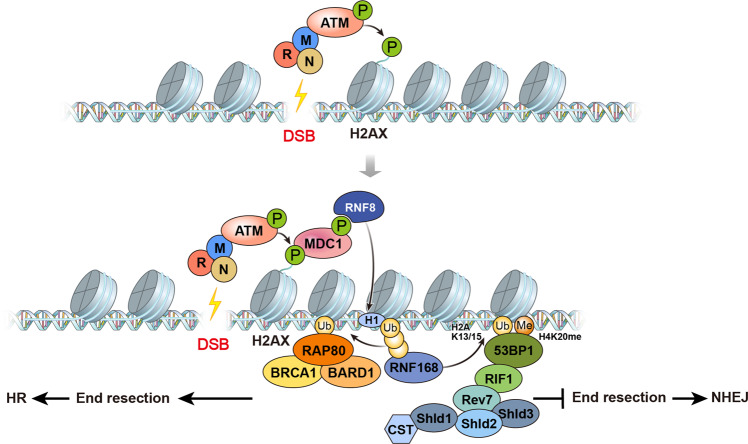
Overview of the DNA damage signaling pathway. Upon DNA damage, DSBs (DNA double-strand breaks) are sensed by the MRN complex and ATM homodimer. ATM is activated by autophosphorylation at S1981 and phosphorylates histone H2AX at S139. Phosphorylated H2AX, called γH2AX, recruits MDC1 and further propagates γH2AX signaling at DSB sites. MDC1 is phosphorylated by ATM, and phosphorylated MDC1 recruits the ubiquitin ligase RNF8. The RNF8-UBC13 complex promotes K63-linked polyubiquitination of H1, which is recognized by RNF168. RNF168 initially monoubiquitinates H2A at K13/15 and amplifies polyubiquitin chains. K13/15-monoubiquitinated H2A and H4K20me2 are recognized by 53BP1 and further recruit RIF1 and the Shieldin complex to promote NHEJ repair. Alternatively, the RNF168-mediated polyubiquitin chain also recruits RAP80 and the BRCA1/BARD1 complex, further promoting HR repair.

The human genome is continuously exposed to endogenous and exogenous inducers of DNA damage; however, the genome is protected against such insults by multiple, interdependent DNA repair pathways collectively known as the DDR^5^. Many DDR signaling and repair factors have been identified, and recently, their functions on the chromatin structure have received much attention (Fig. 1). Upon DNA damage, DSBs are detected by DSB sensors such as the MRE11 endonuclease and recruit NBS1 and Rad50 to form the MRN complex. MRN bound to DNA recruits the ATM kinase, which is subsequently activated by autophosphorylation. Activated ATM phosphorylates histone H2AX at S139 to form ɣH2AX, which allows further recruitment of the mediator protein MDC1 (mediator of DNA damage checkpoint protein 1)^6,7^. MDC1 further enhances the recruitment of the MRN/ATM complex and propagates ɣH2AX up to 1–2 megabase pairs *in cis* to the DSB^8–10^. ɣH2AX propagation recruits RNF8-UBC13, which promotes K63-linked polyubiquitylation of H1, which is in turn recognized by RNF168^11^. RNF168 monoubiquitinates H2A at K13/15 and amplifies K63-linked polyubiquitin chains, generating a platform for repair factors^12,13^. H2A ubiquitination at K13/15 is recognized by the DSB repair protein 53BP1^14^. 53BP1 further recognizes H4K20me2, whose level remains unchanged upon DNA damage. RNF8 and RNF168 indirectly promote 53BP1 recruitment by ubiquitinating H4K20me2-binding factors, L3MBTL1, and trigger the dissociation of L3MBTL1 from chromatin, which unmasks H4K20me2 and allows 53BP1 recruitment^15^. Furthermore, RNF168-mediated K63-linked polyubiquitin chains on histones recruit RAP80 and the BRCA1/BARD1 complex^16–18^. Thus, histone modifications, such as ubiquitination, phosphorylation, and methylation, are crucial to the sequential recruitment of DDR factors and repair factors. DSB repair largely occurs via two major DSB repair pathways: nonhomologous end joining (NHEJ) and homologous recombination (HR). It is accepted that DSB repair pathway choice is dependent on the cell cycle status, with NHEJ driven by 53BP1-RIF1 in the G1 phase^19–22^ and HR driven by BRCA1 in the S/G2 phases, when the homologous template is present. Pathway choice is, in part, determined by DSB end resection^23,24^. Cell cycle-dependent phosphorylation and ubiquitination of CtIP is important for DNA end resection, which favors HR^25,26^. Furthermore, the transcriptional status of chromatin can affect pathway choice^27^. Recently, Nakamura et al. reported that H4K20 methylation levels oscillate during the cell cycle and that changes in H4K20 methylation determine whether BRCA1 or 53BP1 is recruited to DSB sites in pre- or postreplicative cells. During the G1 phase, H4K20me1/2 is recognized by 53BP1, while H4K20me0 newly incorporated in the S phase recruits BRCA1-BARD1 to DSB sites and opposes 53BP1 function^28^. Furthermore, BARD1 interacts with HP1-mediated H3K9me2 in response to DNA damage and retains the BRCA1 complex to promote HR and inhibit NHEJ^29^. From this perspective, chromatin dynamics become more critical in the DNA damage response and DNA repair.

### Transcriptional silencing in the DNA damage response

**Fig. 2:**
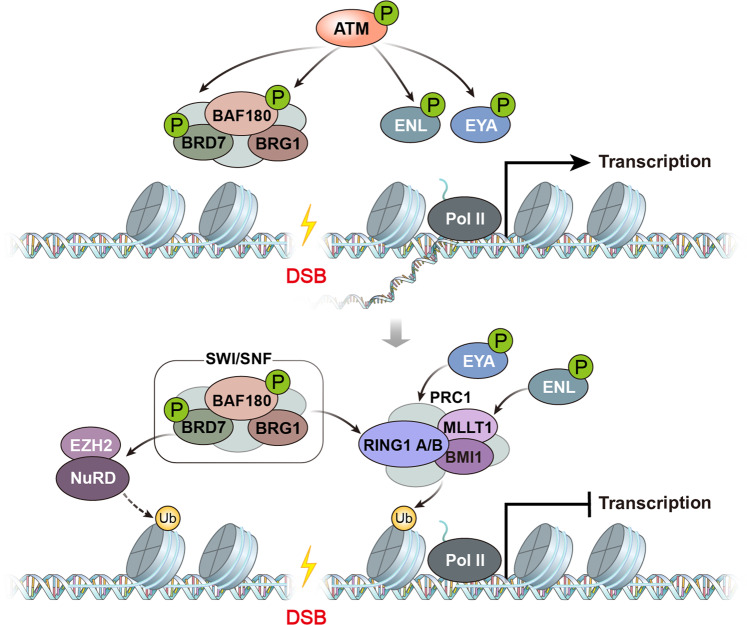
ATM-mediated transcriptional silencing *in cis* to DNA breaks. Activated ATM phosphorylates multiple substrates to silence active transcription *in cis* to DNA breaks. The chromatin remodelers BAF180 and BRD7 are phosphorylated and recruit the PRC1 and PRC2 complexes, respectively. The transcription elongation factor ENL is also phosphorylated by ATM and promotes H2A-K119 ubiquitination. ATM-dependent phosphorylation of EYA is another important player in DSB-induced transcriptional silencing via dephosphorylation of H2AX at Y142.

When DSBs occur at or near an actively transcribed region of chromatin, the transcription of genes *in cis* to the damage site is silenced^30^. The ATM kinase has been proposed as a master regulator of transcription, recruiting polycomb repressive complex (PRC) to halt transcription^2^ (Fig. 2). H2A ubiquitination at K119 by PRC is the canonical histone marker for transcriptional repression. H2A-K119 ubiquitination extends more than 14 kb from the break site^31^, a more dynamic change than that in H3K27me3 mediated by EZH2^32–34^. ATM phosphorylates the transcription elongation factor ENL, allowing its interaction with PRC1^35^ and repressing transcription elongation. EYA3 similarly contributes to transcriptional repression at DNA damage sites upon phosphorylation by ATM/ATR^36,37^. The E3 ubiquitin ligase UBR5 is recruited by PRC1 and interacts with the FACT complex to transiently suppress FACT activity, blocking transcription at UV lesions and DSB sites^38^. Later, the same group reported that the deubiquitinase OTUD5 similarly regulates SPT16 enrichment and blocks Pol II elongation at DSB sites^39^. Chromatin remodeling factors are also involved in transcriptional silencing: both BAF180, a subunit of the PBAF chromatin remodeling complex, and the BRG1 ATPase are required for the recruitment of PRC1^40^. BRD7, another subunit of the PBAF complex, is phosphorylated by the ATM kinase, and this phosphorylated BRD7 interacts with MRN/PRC2, promoting H2A-K119 ubiquitination^41^. Thus, the ATM kinase activates signaling cascades for both DDR and DSB-induced transcriptional silencing by phosphorylating multiple substrates.

**Fig. 3:**
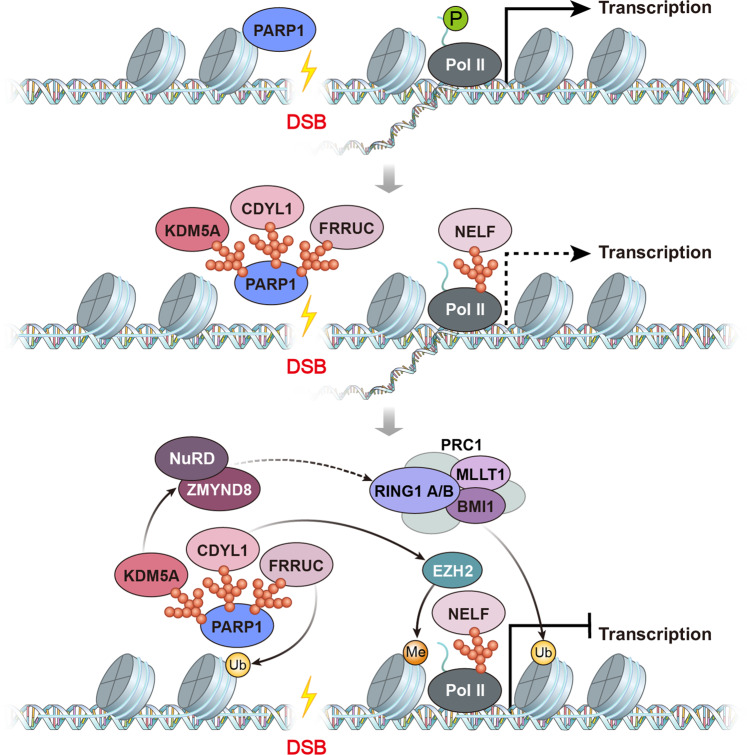
PARP1-mediated transcriptional silencing at DNA breaks. PARP1 PARylates histones and nonhistone proteins and recruits KDM5A, FRRUC, CDYL1, and NELF to DSB sites. PARP1-mediated recruitment of KDM5A in turn recruits the ZMYND8-NuRD complex and promotes transcriptional silencing. FRRUC (FBXL10-RNF68-RNF2 Ubiquitin ligase Complex) is recruited to DSB sites in a PARP-dependent manner and promotes H2A-K119 ubiquitination for transcriptional silencing. RNAPII is PARylated by PARP1 and recruits NELF-E to pause transcriptional elongation. CDYL1 is also recruited to DSB sites by PAR moieties added by PARP1 and recruits EZH2, promoting H3K27me3 at DSB sites for transcriptional silencing.

Poly (ADP-ribose) polymerase 1 (PARP1) is another master regulator of transcriptional silencing at DSBs; this enzyme catalyzes the synthesis of PAR chains on histone and nonhistone proteins at DSBs (Fig. 3)^42,43^. The generation of these negatively charged PAR chains enables the relaxation of chromatin and facilitates the movement of the chromatin remodeler ALC1, which promotes nucleosome eviction at damaged chromatin^44^. PAR chains similarly facilitate the movement of the DDR factors MRE11 and RNF168 to DSB sites^45,46^, while PARylation of RNF168 promotes the recruitment of the chromatin remodeler SMARCA5 (SNF2H) to damaged chromatin. PARP1 activity is also required for the recruitment of PRC and NuRD complexes, which create a transient repressive chromatin structure^47^. PARP1-mediated H2A-K119 ubiquitination appears to be regulated by the noncanonical PRC FRRUC (FBXL10-RNF68-RNF2 ubiquitin ligase complex)^48^. In addition, PARP1 regulates the chromatin remodeling factors ZMYND8 and NuRD^49^. KDM5A-mediated H3K4me3 demethylation recruits the ZMYND8-NuRD complex to damaged chromatin and supports DSB-induced transcriptional silencing^50^. Interestingly, NELF-E acts as a negative transcription elongation factor, moving to DSB sites near transcriptionally active regions in a PARP-dependent manner. The recruitment of NELF-E is important for DSB-induced transcriptional silencing^51^. In addition, the DYRK1B kinase is recruited to DSB sites in a PARP-dependent manner and phosphorylates the histone methyltransferase EHMT2 at T346 to promote transcriptional silencing^52^. Indeed, PARylation at DSB sites is also important for the recruitment of CDYL1, which binds to PAR moieties and recruits EZH2. The recruitment of EZH2 promotes H3K27me3 at DSB sites and, eventually, transcriptional silencing^53^. Thus, both ATM and PARP recruit PRC and multiple chromatin-bound proteins to turn off RNAPII elongation for DSB-induced transcriptional silencing, while PARylation by PARP enzymes at DSB sites remodels the chromatin landscape to allow transcriptional repressors to bind. In addition, RNAPII is directly regulated by proteasomal degradation at DSB sites in a manner mediated by DNA-PK. Upon DNA-PK inhibition, RNAPII bypasses DNA breaks and remains at transcribed loci for transcriptional elongation^54^. Recently, WWP2 was identified as an RNAPII-degrading E3 ligase, synthesizing K48-linked ubiquitin chains^55^. Recently, Steurer et al. reported that genome-wide degradation of promoter-bound RNAPII is mediated by VCP proteosomal degradation after UV irradiation^56^. Thus, degradation of RNAPII upon DNA damage may be the subsequent event after stalling of RNAPII at actively transcribed loci.

Lannelli et al. reported transcriptional repression following the induction of endogenous DNA damage in the DIvA cell line. This group used multiple approaches to monitor the transcription status after DNA damage and found that transcription is highly repressed at the sites proximal to DSBs, and this repression decreases upon movement away from the lesion^57^. However, ChIP-seq showed greater enrichment of RNAPII at sites of damage, while the γH2AX density was decreased at sites of DNA damage, suggesting that DNA damage-induced transcription, such as the transcription of DNA damage response RNAs (DDRNAs) and damage-induced long noncoding RNAs (dilncRNAs), is ongoing at sites of DNA damage. These RNAs are known to recruit DDR factors and form DDR foci^57^. Recently, the same group reported that a preinitiation complex (PIC) is formed at sites of DSBs to allow DDRNA transcription and that RNA synthesis at DSBs is an important step in the formation of 53BP1 condensates by liquid‒liquid phase separation^58^. Thus, preexisting transcription is coordinately regulated upon DNA damage. To prevent collision between these two machineries, cells typically pause the transcription of coding RNAs at DSB sites by turning off active RNAPII at the gene locus until the DNA damage is completely repaired. Meanwhile, active transcription of ncRNAs at sites of DNA damage promotes the recruitment of DDR factors and efficient DNA repair.

### Transcription-coupled DSB repair

We have discussed the crosstalk between the RNAPII machinery and DDR factors. This regulatory mechanism prevents aberrant transcription and improper DNA repair at actively transcribed regions and maintains genomic integrity. Transcription-coupled repair of DNA damage is well documented, especially during repair of UV-induced damage. DNA damage by UV induces 6–4 photoproducts and cyclopyrimidine dimers, which are mainly repaired by nucleotide excision repair (NER). At transcribed loci, the transcription machinery can assist in the repair of UV-induced damage by so-called transcription-coupled nucleotide excision repair (TC-NER). This repair pathway is critical for the removal and repair of UV lesions and to prevent the occurrence of mutations in coding regions^59^.

Transcription-coupled DSB repair was first proposed in yeast, when it was observed that DSBs within active genes were repaired more quickly than DSBs in inactive genes^60^. Later, Wei et al. showed that transcription-coupled DSB repair specifically occurs during the G0/G1 phase in mammals^61^. HR factors such as RAD51C, RAD52, and RPA1 are recruited to transcriptionally active damage sites, and this recruitment is highly dependent on CSB, one of the factors required for TC-NER. Furthermore, the repair of ROS-induced strand breaks is mediated by actively transcribed RNA, which is required for retention of the HR factor RAD52. The interaction between CSB and RAD52 in response to ionizing radiation depends on the presence of an RNA transcript. These events occur primarily in the G0/G1 phase but not in the S phase, which suggests that RNA transcripts can be used as a template for HR in nonreplicating or postmitotic cells^61^. In addition, there is crosstalk between transcription machineries and HR factors at DSBs located within actively transcribed DNA. More recently, it has been shown that CSB recognizes R-loops at transcribed loci, with ROS-induced R-loops recruiting RAD52 and RAD51 through a noncanonical BRCA1/2-independent HR pathway^62^. R loops at transcriptionally active sites recognized by Rad52 also recruit XPG and BRCA1 and antagonize RIF1-53BP1 complex formation in the G2 phase^63^. In the G2 phase, BLM is preferentially recruited to DSBs at transcriptionally active genes and contributes to end resection and HR repair^64^. These data suggest that DSBs in actively transcribed regions of the genome require a unique repair mechanism as a result of the presence of both the transcription and DNA repair machineries at damaged loci.

### Chromatin-based DSB repair

Another layer of the regulatory mechanism invoked upon DNA damage at actively transcribed loci is the chromatin-based choice of DSB repair pathway. Histone modifiers can act as decision-makers, decorating chromatin to block or allow transcription. The Legube group has studied chromatin-based DSB repair using the DIvA cell line, in which endogenous DNA damage can be induced at transcriptionally active or inactive loci. Aymard et al. showed that DSBs in transcriptionally active chromatin undergo resection, recruiting the HR factor Rad51, and these events depend on preexisting histone marks associated with active transcription, such as H3K36me3^65^. The chromatin-based DSB repair pathway choice was further demonstrated by comprehensive mapping of histone modifications at DSB sites using ChIP-seq. Chromatin environments were found to influence the choice between HR and NHEJ, showing the importance of chromatin in DSB repair^66^.

At transcriptionally active loci, DSBs cluster mainly during the G1 phase and delay repair. High-throughput genome-wide sequencing revealed DSB clusters after DSB induction during the G1 phase, and this clustering required the MRN complex and LINC complex, delaying repair by NHEJ and favoring repair by HR. Thus, actively transcribed chromatin favors HR repair, recruiting Rad51 in the S phase, which allows the transcribed chromatin to be repaired by the error-free HR repair pathway in the S and G2 phases^67^. Thus, chromatin architecture plays an important role in the choice of DSB repair pathway and maintains genomic stability at actively transcribed regions.

### Chromatin remodeling factors in the DNA damage response at actively transcribed loci

**Fig. 4:**
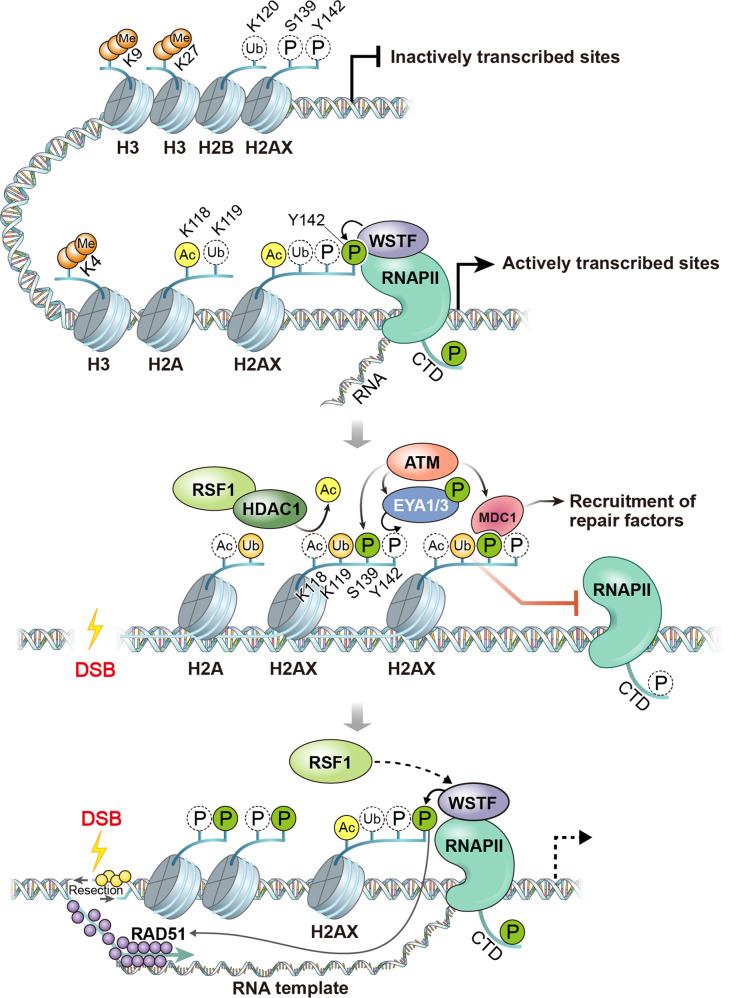
ISWI chromatin remodeling complexes in DSB-induced transcriptional silencing and repair. Histone modifications such as H2AX Y142 phosphorylation and H2A(X) K118 acetylation are enriched at actively transcribed regions. These histone modifications are regulated upon DNA damage. H2AX Y142 is dephosphorylated by the ATM-mediated EYA phosphatase and promotes DSB-induced transcriptional silencing. H2A(X) K118 acetylation is removed at DSB sites by the RSF1-HDAC1 complex to silence active transcription to support DSB repair. Deacetylation of H2A(X) at K118 promotes the ubiquitylation of H2A(X) at K119 by RNF2 for DSB-induced transcriptional silencing. Deacetylation of H2A(X) at K118 and ubiquitylation of H2A(X) at K119 amplify γH2AX signaling at DSB sites and recruit MDC1 for propagation of DNA damage signaling in the G1 phase. After DNA breaks are repaired by repair factors, WSTF rephosphorylates H2AX at Y142 and activates transcription and RNA-templated DSB repair by recruiting RAD51 in the G1 phase.

Chromatin context and architecture are established by chromatin remodeling complexes and histone modifiers. Chromatin remodeling factors were initially identified as transcriptional regulators that compact or loosen chromatin during transcription. Four families of chromatin remodeling factors have been identified in mammals: SWI/SNF, ISWI, NuRD and INO80. Chromatin remodeling by these factors similarly affects chromatin dynamics upon DNA damage^68^. Most chromatin remodeling factors are involved in DSB repair, especially in the propagation of ɣH2AX^69^. Chromatin compaction/relaxation is critical for the recruitment of DSB repair factors and activation of the DDR^70^. The ATM kinase regulates chromatin relaxation to relay the DSB signal and silence transcription *in cis* to DSB sites^30^. Chromatin remodeling factors also regulate chromatin architecture, which controls ongoing transcription at DSB sites. The PBAF complex, a member of the SWI/SNF family, was the first chromatin remodeling factor identified that silenced active transcription upon DNA damage. The PBAF complex subunit BAF180, in the SWI/SNF family, is phosphorylated by ATM and recruits PRC1 and PRC2 for DSB-induced transcriptional silencing and DSB repair^40^. Later, the same group reported that cohesin, in cooperation with PBAF, represses transcription near DSBs^71^. Another subunit in the PBAF complex, BRD7, is phosphorylated by ATM and recruits the chromatin remodeling complex NuRD^41^. The subunits in the NuRD complex, such as HDAC1, normally repress transcription; thus, the recruitment of the NuRD complex may have pivotal roles in subsequent transcriptional silencing at transcribed loci^72^. Recently, ISWI chromatin remodeling complexes were found to be critical for DSB-induced transcriptional silencing^37,73^ (Fig. 4). The regulatory subunits of these ISWI complexes, ACF1, WSTF, and RSF1, have distinct roles in transcription and DNA damage, while they all share a common ATPase subunit, SNF2H. WSTF has kinase activity to phosphorylate H2AX at Y142. Upon DNA damage, H2AX-pY142 is dephosphorylated, while ɣH2AX is propagated. Cook et al. reported that dephosphorylation of H2AX-pY142 by the EYA1/3 phosphatase is a prerequisite for ɣH2AX propagation and that this signaling is important for the cell fate decision between survival and apoptosis^36^. A recent study emphasized the importance of WSTF-induced H2AX-pY142 as a regulatory switch to control transcription upon DNA damage at transcriptionally active loci. Ji et al. identified that H2AX-pY142 interacts with RNAPII in proliferating cells and that its dephosphorylation by ATM-dependent EYAs upon DNA damage disrupts this interaction, silencing ongoing transcription. During DNA repair, WSTF rephosphorylates H2AX-Y142, and this *de novo* phosphorylation recruits RAD51 to activate transcription-coupled HR (TC-HR) in the G1 phase^37^. Thus, the chromatin remodeler WSTF fine-tunes transcription and promotes TC-HR in the G1 phase to maintain genomic integrity.

Remodeling and Spacing Factor 1 (RSF1) is an ISWI family member that interacts with the SNF2h ATPase. RSF1 is phosphorylated by the ATM kinase and regulates DNA damage signaling and DSB repair^74–76^, interacting with ATM to regulate HR and NHEJ^77^. RSF1 also plays an important role in mitosis to maintain appropriate segregation of chromosomes and genomic integrity^78–80^. RSF1 deficiency in neural-specific RSF1 knockout mice resulted in normal brain development; however, apoptosis triggered by exogenous DNA strand breaks during neurogenesis was reduced. This was further supported by RNA sequencing upon DNA damage in an RSF1 KO cell line. RNA-seq analysis showed a reduction in p53-mediated transcription in the absence of RSF1, resulting in impaired binding of p300 to the promoters of p53 target genes such as p21 and the proapoptotic genes PUMA and BAX. This appeared to be driven by a reduction in H3 acetylation on the promoters of p53 target genes, which may have altered the chromatin environment and induced transcriptional reprogramming in RSF1 KO cells^81^. Furthermore, mass spectrometry showed that RSF1 interacts with many histone modifiers, such as EZH2 and HDAC1. The RSF1-HDAC1 interaction regulates deacetylation at mitotic centromeres and facilitates phosphorylation of H2A at T120 to allow correct segregation of chromosomes during mitosis^79^. This interaction is important in response to DNA damage at transcribed loci. Upon DNA damage, H2A-K119 ubiquitination by PRC is a cue for transcriptional silencing at DSBs. The RSF1-HDAC1 complex deacetylates H2A-K118ac, which is enriched at sites of active transcription, to allow efficient ubiquitination of H2A at K119, in turn inducing transcriptional silencing. This temporal regulation of H2A-K118 deacetylation and H2A-K119 ubiquitination mediates crosstalk between transcription and the DDR, further promoting ɣH2AX propagation and DSB repair^73^. Preexisting histone modifications, such as H2AX-pY142 and H2A(X)-K118ac, in transcribed chromatin are regulated by ISWI family members to allow transcriptional repression *in cis* to DNA damage for efficient DNA repair by ɣH2AX propagation. Inhibition of transcription may promote RNAPII pausing at DSB sites, thus promoting R-loop formation to allow efficient RNA template-mediated HR repair by recruiting HR factors, such as RAD51^82^. This regulatory mechanism, driven by chromatin remodelers, also emphasizes the importance of preexisting chromatin in influencing DSB repair pathway choices^27^.

ISWI has been identified to primarily deposit and slide nucleosomes to create regular spacing^68^. Critical nuclear events, such as DNA damage, require rapid signal transduction to regulate cellular events, such as DNA transcription. To ensure rapid signal transduction, cells may evolutionarily acquire nucleosome modifications by rapidly recruiting histone-modifying enzymes and chromatin remodelers to actively transcribed loci rather than by exchanging nucleosomes. p400, an ATPase in the INO80 family, has been shown to exchange histone H2A with the H2A variant H2A.Z to allow chromatin relaxation and efficient repair^83,84^. The primary roles of chromatin remodelers, such as nucleosome eviction and chromatin relaxation, may become critical in heterochromatin upon DNA damage. Thus, chromatin remodelers are likely to moderate chromatin environments by recruiting histone modifiers, and the spatial and temporal regulation of histone modifiers by chromatin remodelers may govern genomic integrity following various cellular events. Chromatin modification by each chromatin remodeler may play a selective role upon DNA damage, and the specific function of each chromatin remodeler needs to be investigated further.

## Concluding remarks

In this review, we summarized the regulatory mechanisms of the DDR and DNA repair at actively transcribed loci. DNA DSBs in actively transcribed chromatin need particular attention to prevent aberrant RNA transcript production and allow efficient, error-free DNA repair. Chromatin dynamics play an important regulatory role in preventing collision between the DNA repair and transcription machineries, leading to accurate DNA repair. Specific histone modifications establish the chromatin environment and recruit chromatin remodeling factors and histone modifiers to modulate chromatin dynamics. Upon DNA damage, a chromatin transition may be required, depending on the chromatin environment, and it can aid in the selection of a DSB repair pathway that is favorable for accurate DSB repair. Advanced NGS techniques have allowed researchers to visualize chromatin changes following treatment with various stimuli and to analyze the chromatin-associated factors involved in these changes. Hi-C techniques have also unveiled the chromatin architecture under certain cellular conditions.

Chromatin versatility is spatially and temporally controlled by chromatin remodelers that may read the status of chromatin to allow them to recruit appropriate histone modifiers at the right time and the right place. However, how specificity is granted to these abundant chromatin remodelers is currently unknown. In addition, further study into how chromatin remodeling factors and histone modifiers deal directly with RNA in R-loops and chromatin clusters is required to show how chromatin architecture, together with the regulation of chromatin dynamics, drives DNA repair at actively transcribed loci.
